# Mental, Physical and Social Functioning in Independently Living Senior House Residents and Community-Dwelling Older Adults

**DOI:** 10.3390/ijerph182312299

**Published:** 2021-11-23

**Authors:** Anna-Maria Lahti, Tuija M. Mikkola, Minna Salonen, Niko Wasenius, Anneli Sarvimäki, Johan G. Eriksson, Mikaela B. von Bonsdorff

**Affiliations:** 1 Gerontology Research Center and Faculty of Sport and Health Sciences, University of Jyväskylä, 40014 Jyväskylä, Finland; Mikaela.vonbonsdorff@jyu.fi; 2 Folkhälsan Research Center, 00250 Helsinki, Finland; tuija.mikkola@folkhalsan.fi (T.M.M.); minna.salonen@thl.fi (M.S.); niko.wasenius@helsinki.fi (N.W.); johan.eriksson@helsinki.fi (J.G.E.); 3 Clinicum, Faculty of Medicine, University of Helsinki, 00014 Helsinki, Finland; 4 Public Health Promotion Unit, National Institute for Health and Welfare, 00271 Helsinki, Finland; 5 Department of General Practice and Primary Health Care, University of Helsinki and Helsinki University Hospital, 00014 Helsinki, Finland; 6 The Age Institute, 00520 Helsinki, Finland; Anneli.sarvimaki@ikainstituutti.fi; 7 Singapore Institute for Clinical Sciences, Agency for Science, Technology, and Research, Singapore 117609, Singapore; 8 Department of Obstetrics & Gynaecology and Human Potential Translational Research Programme, Yong Loo Lin School of Medicine, National University of Singapore, Singapore 119077, Singapore

**Keywords:** mental functioning, older people, physical functioning, loneliness, senior housing, social contacts

## Abstract

Senior houses provide social interaction and support, potentially supporting older people’s physical and mental functioning. Few studies have investigated functioning of senior house residents. The aim was to compare functioning between senior house residents and community-dwelling older adults in Finland. We compared senior house residents (*n* = 336, 69% women, mean age 83 years) to community-dwelling older adults (*n* = 1139, 56% women, mean age 74 years). Physical and mental functioning were assessed using the SF 36-Item Health Survey. Loneliness and frequency of social contacts were self-reported. The analyses were adjusted for age, socioeconomic factors and diseases. Physical functioning was lower among men in senior houses compared to community-dwelling men (mean 41.1 vs. 46.4, *p* = 0.003). Mental functioning or the frequency of social contacts did not differ between type of residence in either sex. Loneliness was higher among women in senior houses compared to community-dwelling women (OR = 1.67, *p* = 0.027). This was not observed in men. Results suggest that women in senior houses had similar physical and mental functioning compared to community-dwelling women. Male senior house residents had poorer physical functioning compared to community-dwelling men. Women living in senior houses were lonelier than community-dwelling women despite the social environment.

## 1. Introduction

The population is aging rapidly in Western countries. Aging increases risks of an overall decline in health, and aging of the population results in an ever-growing demand for suitable housing options. Because younger generations’ functional ability is better than before [1,2], there is likely to be an increasing need for non-institutional housing facilities that provide light services and sense of community to support independent living. Finland provides an interesting setting for our study, as Finland is a Nordic welfare state with free or low-cost healthcare. In the Nordic welfare model, healthcare is available to everyone regardless of financial resources [3].

Senior houses, i.e., non-institutional facilities, where older people live independently, constitute one potential form of living for older adults who have varying needs for assistance in daily living tasks. While care for older people is mainly organised at the municipal level in Finland, senior houses and other independent living facilities are usually privately owned or rental apartments owned by non-profit organisations and foundations. Senior houses are often targeted for people aged 55 years or older who are able to live independently. The residents pay for rent and various services offered by the facilities, such as housekeeping and food service, to support their independent living. Unlike assisted living facilities and nursing homes, senior houses do not provide continuous care or assistance. Senior housing has become increasingly popular in the recent years [4]. Based on previous international research, reasons for relocating to a senior housing facility often include decline in physical performance, lack of assistance and loneliness [5,6] but also a desire for a safer and more comfortable living environment [6,7].

Senior houses often have organised activities and common spaces. Thus, the facilities provide opportunities for social interactions and hence, may provide social support [8]. Neighborhood social capital and social support have been associated with better mental health among older individuals [9], and social participation and a wider social network have been associated with better physical health and lower mortality [10,11,12]. A recent qualitative study found that senior housing offered an environment that supports well-being and healthy aging [13]. These findings support the idea that senior houses could promote mental and physical functioning of older people.

Loneliness is a common and growing problem among older adults, and it has been associated with several negative physical and mental health outcomes, such as decline in functioning [14] and self-rated health [15], depression, cognition and premature mortality [16,17,18]. Residents in senior houses could have more frequent social contacts due to the surrounding social environment. Having peers as neighbors and activities organized in the houses are factors that may contribute to social interaction and thus alleviate or even prevent loneliness [8,19], although the frequency of social contacts alone does not necessarily indicate less loneliness [20]. In fact, a longitudinal study on mental wellbeing of residents in senior housing reported that loneliness did not change during the first year of living in senior housing [21]. Interestingly, a previous study on the social environment in senior housing found that the residents’ social contacts increased while living in senior housing [22].

Less is known of the functioning and characteristics of senior house residents in relation to community-dwelling older people. Most studies of functioning and loneliness have been conducted among community-dwelling older people, and not specifically in an independent-living senior house setting. In addition, only a few studies have compared senior house residents and community-dwelling older adults [6,23]. A recent cross-sectional study on health of independently living residents of a senior housing community found no differences in physical or mental well-being between the residents and community-dwelling older people [6]. However, another study reported that residents in a senior housing community reported better self-rated health than community-dwelling older people [23]. Findings by Taylor et al. [19] and Jeste et al. [6] suggest that residents in senior housing communities seem to be lonelier than the general population, despite the social environment.

The aim of this cross-sectional study was to contribute to the limited knowledge of independent senior housing by investigating whether independently living male and female senior house residents’ physical, mental and social functioning as well as loneliness differed from community-dwelling older adults’ functioning and loneliness. We expected that (1) physical functioning would be similar to or better in senior house residents compared to community-dwelling older adults; (2) mental functioning would be similar to or better in senior house residents compared to community-dwelling older adults; (3) senior house residents would be more lonely compared to community-dwelling older adults; and (4) senior house residents would have more frequent social contacts than community-dwelling older adults.

## 2. Materials and Methods

We used data from the BoAktiv senior housing study and self-reported survey data from the Helsinki Birth Cohort Study (HBCS). In total, our study included 1475 individuals. BoAktiv data were collected in 2018 and in 2020 in 15 senior houses around coastal regions of Finland. All residents who were over 55 years of age, Finnish- or Swedish-speaking and living independently were invited to take part in the study by filling in a survey at home delivered by the researchers. In 2018, we invited a total of 465 persons of which 194 participated (41.7%). In 2020, we included three new senior houses that had been recently opened and invited 588 individuals of which 247 participated (42.0%). Altogether, 105 individuals answered the survey both in 2018 and 2020 and in these cases answers from 2018 were included. Thus, the number of eligible subjects in 2020 was 142 resulting in a total sample size of 336 participants. They are herein referred to as ‘senior house residents’.

We used data from the Helsinki Birth Cohorts Study (HBCS) to obtain a group of community-dwelling older adults. HBCS is an ongoing epidemiological longitudinal cohort study, which includes 13,345 individuals born between 1934 and 1944 in Helsinki, Finland at the Helsinki University Central Hospital or Helsinki City Maternity Hospital. In 2000, a sample of 2691 individuals were invited to participate in a clinical examination between 2001 and 2004 using random-number tables. Of these individuals, 2003 cohort members participated in clinical examinations and interviews conducted by trained study nurses as described in [24,25]. Of these individuals, a postal questionnaire was sent out in 2015 to the cohort members who were alive and whose contact information was available (*n* = 1577). Of them, 1153 cohort members participated, which is the sample used in this study. The cohort members were asked in 2015 about their living conditions and those living in a senior house or a service home (*n* = 14) were excluded from the study and all other cohort members were included in the community-dwelling study cohort (*n* = 1139). These cohort members of the HBCS are herein after referred to as ’community-dwelling older adults’.

Both studies were conducted in line with the guidelines of the Declaration of Helsinki. BoAktiv was approved by the University of Helsinki Ethical Review Board in the Humanities and Social and Behavioral Sciences, and the HBCS was approved by the Ethics Committee of Epidemiology and Public Health of the Hospital District of Helsinki and Uusimaa and the National Public Health Institute, Helsinki. Participants in both studies received a description of the study and provided written informed consent.

### 2.1. Measures

#### 2.1.1. Physical and Mental Functioning

Self-reported physical and mental functioning were assessed using the validated Finnish version of the SF-36 Health Survey 1.0 [26,27], a widely-used method for assessing health. The survey consists of eight domains: physical functioning (10 items), role limitations caused by physical health problems (4 items), bodily pain (2 items), general health (5 items), role limitations caused by emotional problems (3 items), vitality (4 items), mental health (5 items) and social functioning (2 items). The score range for each item is from 0 to 100, with 100 representing the best level of functioning or well-being. The eight domains were standardised using the means and standard deviations of the US reference population (1990) [28]. Physical (PCS) and mental component scores (MCS) were calculated from standardised domains. The domains were weighted using factor score coefficients from the same reference population and summed, resulting in PCS and MCS. Finally, PCS and MCS were standardised using a mean of 50 and a standard deviation of 10 [29]. Of the community-dwelling older adults, 1113 (97.7%) had complete data on SF-36 and of the senior house residents 290 (86.3%) had complete data on SF-36.

#### 2.1.2. Loneliness

Using questionnaires, the participants in both studies were asked a standard question: “Do you feel lonely?” and alternatives to the answers were: (1) Never or rarely, (2) Sometimes and (3) Often. As only 4% of the participants reported to have felt lonely often, we combined options 2 and 3 into one group, forming the group ‘lonely’.

#### 2.1.3. Social Functioning

Social functioning was measured using the frequency of social contacts. The participants in both studies were asked “How often are you in contact with your family or children/close friends/acquaintances?” using three separate questions. Options to the answers were: (1) Every day, (2) Every week, (3) Every month, (4) Couple of times a year, (5) Seldom or not at all and (6) I don’t have family/close friends/acquaintances. Answer options were recoded into: (1) Every day, (2) Weekly or monthly and (3) Rarely or not at all. Due to small frequencies in the categories, the questions on family and close friends were combined by summing the variables and recoding them into a similar three class-scale as was done for contact with acquaintances.

#### 2.1.4. Covariates

Sociodemographic and socioeconomic variables include age, gender, marital status (1) Married or in a relationship, (2) Non-married, education (1) University level, (2) High school (3) Middle school or lower, economic situation (1) Very good or good, (2) Average, (3) Poor or very poor, alcohol intake (1) Weekly, (2) Monthly or less, (3) Not at all smoking (1) Smoking, (2) Not smoking, physical activity (times per week) and chronic diseases. Medical history of 11 chronic illnesses were self-reported in both studies, including heart insufficiency, angina pectoris, myocardial infarction, heart arrythmia, stroke, cancer, diabetes, dementia, chronic pulmonary diseases (e.g., asthma), claudication and rheumatic disorders.

### 2.2. Statistical Methods

Baseline characteristics were compared using Mann-Whitney’s U-test to compare the mean scores of continuous variables since many of the distributions were not normal. The chi-squared (χ^2^) test was used for categorical variables, and Fisher’s exact test was used for dichotomous categorical variables. All tests were conducted separately for women and men, since there may be significant differences in health between the sexes [30]. To further compare physical and mental functioning between the housing groups, we used general linear models (GLM) with bootstrapping. The analyses were adjusted for age and socioeconomic factors (marital status, education and economic situation). To compare loneliness among participants, we used binary logistic regression analysis and to compare social contacts, we used ordinal logistic regression analysis. The analyses were adjusted for age, socioeconomic factors and chronic diseases. All tests were performed two-tailed, and analyses were carried out using SPSS IBM version 25.0 (IBM Corp, Armonk, NY, USA) and Stata 15.1 (StataCorp, College Station, TX, USA).

In order to control for the age difference in the two cohorts and to exclude outliers, we ran the analyses including only those senior house residents who were within the age range of the community-dwelling older people (born between 1934 and 1944). We found that the results were very similar and had the same trend for both physical and mental functioning, loneliness and social contacts as in the non-stratified analyses. Here, we report only analyses including all senior house residents.

## 3. Results

### 3.1. Participant Characteristics

Of the senior house residents, 69% were women. Of the community-dwelling older adults, 56% were women. The mean age for senior house residents was 83 years (±7.6) and for community-dwelling older adults 74 (±2.7) years. Senior house residents differed from the community-dwelling older adults in various demographic and health variables (Table 1). A larger percentage of senior house residents were not married or in a relationship. A larger proportion of them also had a university level degree, and they had more chronic diseases compared to community-dwelling peers. Senior house residents used less alcohol and fewer of them smoked, and they were less physically active.

### 3.2. Health-Related Quality of Life SF-36

Senior house residents had lower health-related quality of life in all of the eight SF-36 subscales compared to older people living in the community, except for bodily pain (data not shown). The associations between housing type and health-related quality of life are presented in Table 2. After adjusting for age there were no marked differences in the SF-36 subscales among women, and in most subscales among men, apart from physical function, which was 12.9 (*p* = 0.001) points lower among senior house residents, and social function, which was 6.8 (*p* = 0.019) points lower among senior house residents. When adjusting for age and socioeconomic factors, the differences among men in physical function increased to 18.3 points (*p* < 0.001) and in social function to 9.8 points (*p* = 0.002). No marked differences were found among women.

### 3.3. Mental Functioning

**Figure 1:**
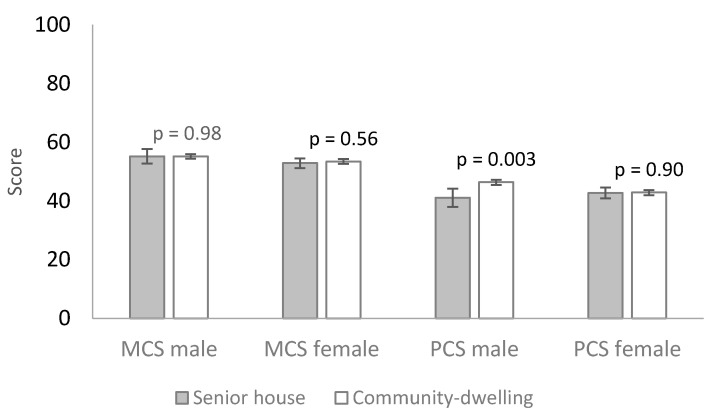
The SF-36 physical and mental functioning summary scores for males and females in housing groups using General Linear Model (GLM) adjusted for age, educational attainment, marital status and economic situation. MCS = mental component summary score, PCS = physical component summary score.

MCS was lower among senior house residents. The difference was small, 1.9 (*p* = 0.06) points among men and 3.1 (*p* < 0.001) points among women (Table 1). Further adjustment for age and socioeconomic factors attenuated the differences in the MCS according to housing type among men and women, presented in Table 2 and Figure 1.

### 3.4. Physical Functioning

PCS was lower among senior house residents. Among men, the differences was 5.8 (*p* < 0.001) points and among women, 3.5 (*p* < 0.001) points (Table 1). Adjustment for age attenuated the differences. However, further adjustment for socioeconomic factors strengthened the association among men, 5.3 points (*p* = 0.003) lower among senior house residents indicating negative confounding (Table 2 and Figure 1).

### 3.5. Loneliness

A larger proportion of senior house residents felt lonely at least sometimes compared with older people who lived in the community (Table 1). Among women, the odds for feeling lonely at least sometimes was higher among the senior house residents compared to the community-dwelling older people (Table 3). Further adjustment for age, socioeconomic factors and chronic diseases did not markedly change the association. Among men, there was no association between the housing groups and loneliness.

### 3.6. Social Contacts

Among women, senior house residents were in contact with acquaintances more often than community-dwelling individuals (Table 1). However, we found no differences in the odds ratios for housing groups and contacts with acquaintances in either women or men when adjusted for age, socioeconomic factors and diseases (Table 3). Similarly, housing type was not associated with social contacts defined as contacts with family and close friends.

## 4. Discussion

Findings from this cross-sectional study indicate that older men living in senior houses had lower physical functioning compared to older men living in the community. Among women, the housing type was not associated with physical functioning. In addition, mental functioning was not associated with housing type. Loneliness appeared to be more common in senior houses even though the frequency of social contacts did not differ between the housing groups.

Our findings showed that men in senior houses had lower physical functioning than community-dwelling men. The PCS difference of 5.3 points can be considered as clinically meaningful [31,32]. Our study findings are partly in accordance with a recent study [6] in which the investigators did not find differences in physical functioning between older adults living in senior housing and in the community. However, PCS mean scores were slightly lower in their study compared to ours. In addition, they did not look into sex differences in their study. Another study focusing on the use of health and medical services reported that older adults living in senior housing had better self-rated health than older adults living in the community [23]. It is possible that one of the main reasons an older person chooses to move to a senior house is due to reduced physical functioning, which would explain the differences we observed among men. Women may reside in senior housing for different reasons, such as loneliness. However, as stated in our inclusion criteria, the senior house residents were all living independently. Independent living requires the individual to be in somewhat good overall health.

As expected, mental functioning did not differ between senior house residents and community-dwelling older people. Senior houses offer opportunities for socialisation and intellectual activities, which may have a positive effect on mental health. In addition, senior houses are often centrally located or have good access to public transport and services, which have been associated with increased well-being [9] and better cognitive functioning [33]. Our results are in accordance with results from another study [6] reporting no differences among mental health measured with SF-36 and very similar MCS mean scores as in our study. However, adults choosing to move to senior housing are often well-educated and/or have a good economic situation, which are both associated with better mental health and self-rated health [34] and may explain their good mental functioning. Our results support this idea, since the residents in senior houses were mostly highly educated. The participant characteristics in Finnish senior houses were similar to previous studies in senior communities in terms of sex [35,36,37,38], marital status, educational attainment [6,37] and chronic diseases [23].

We found that residents in senior houses were lonelier than older adults in the community, but after adjustments, this was only observed among women. To our knowledge, few studies have investigated loneliness in a similar senior house setting as in our study where residents live independently. Nevertheless, our findings are similar to another study focusing on loneliness in senior housing [19]. As previously stated, women may reside in senior houses for different reasons than men, such as loneliness or lack of social support. In addition, senior housing is often considered a safe housing option [39] which may be appealing to lonely women in particular. Also, since the senior house residents in our study were older than the community-dwelling older adults, they may have been more likely to have experienced a loss of family and friends during their lifetime. This might be the case especially for women, since a larger percentage of them were widowed. Losing one’s spouse may create a sense of bereavement that we were not able to observe in our study. Bereavement may create a strong feeling of loneliness and sorrow that cannot be reduced with social gatherings [40]. This however may be affected by the amount of time passed since the loss of one’s spouse. It may also be plausible that people who move to a senior house may have greater social needs to begin with, thus being more prone to feeling lonely if their social needs are not met. Furthermore, people who enjoy solitude may be less inclined to consider residing in a senior house in the first place.

The frequency of social contacts did not differ between the housing groups. However, we do not know how satisfied the participants were with the frequency and/or quality of contacts with their family and friends or acquaintances. Loneliness has been associated with the satisfaction of social contacts rather than the frequency [20], which would explain the differences we found in loneliness but not in social contacts. Another issue to consider is the effect of language. Most of the senior house residents were Swedish-speaking, and a previous study has suggested that the Swedish-speaking minority in Finland appear to have greater social capital than the general Finnish-speaking population [41,42] which could explain why we did not observe differences in the amount of social contacts.

The strengths of our study include the use of the SF-36 survey, which is a standardised, validated and commonly used measure, thus allowing our results to be compared to other studies. The SF-36 has been found to be a suitable and valid instrument for use among older people [43,44]. We were also to take functioning into account from not only physical and mental but also from a social perspective. In addition, our sample size was of reasonable size. However, this study has limitations, and the results must be interpreted with caution. Firstly, the participants in the BoAktiv study were living in private senior houses in coastal regions of Finland, and a majority of them lived in the capital region, which is why the generalisability of our study to other senior houses may be limited, since the residents may have different characteristics in the northern and eastern regions of the country. Second, senior house residents were older at the time of answering the survey. However, when we restricted the sample of senior house residents to match the age range of the community-dwelling sample, the results were similar to the analyses including all senior house residents. Hence, we believe that the effect of difference in age between the study groups is unlikely to have a marked effect. Thirdly, we do not have information on the participants’ cognition. Loneliness is associated with cognitive decline, which may impact functioning [45,46]. However, women did not differ regarding functioning among the groups, even though they were more lonely in senior houses. Finally, we lack information of the non-responders. As often occurs in studies of older adults, the individuals with functional decline or more chronic illnesses may not respond to surveys, resulting in better physical functioning than in the population on average.

## 5. Conclusions

The aim of this study was to contribute to the literature by investigating whether independently living senior house residents’ physical, mental and social functioning as well as loneliness differ from those of community-dwelling older adults. Based on our results, female senior house residents in Finland’s coastal regions appear to be somewhat similar to older women living in the community in regard to self-rated physical and mental health. Older men living in senior housing have lower physical functioning than community-dwelling men. Loneliness is nevertheless a problem in senior houses, especially among women, despite the social environment. The results of the present study add to the limited knowledge of this topic and may be of help in developing and planning of senior houses in the future that meet the social needs of residents, since there will be a need for a wide range of housing options that support independent living for as long as possible. Longitudinal studies are needed to further investigate the topic.

## Figures and Tables

**Table 1:** Participants’ demographic and socioeconomic characteristics.

	Men			Women		
Variables	Senior House	Community-Dwelling		Senior House	Community-Dwelling	
			*p*-Value *			*p*-Value *
Age, mean (SD)	82.6 (7.7)	73.9 (2.6)	<0.001	83.1 (7.6)	74.0 (2.8)	<0.001
Married or in a relationship, *n (%)*	55 (52)	419 (84)	<0.001	45 (20)	368 (58)	<0.001
Education, *n (%)*			<0.001			<0.001
University level	60 (58)	137 (28)		100 (44)	133 (21)	
High school	23 (22)	253 (51)		76 (34)	288 (45)	
Basic education	20 (19)	108 (22)		51 (23)	217 (34)	
Economic situation, *n (%)*			0.44			0.80
Good or very good	70 (67)	266 (53)		110 (49)	306 (48)	
Mediocre	33 (31)	215 (43)		97 (43)	287 (45)	
Poor or very poor	<5 (2)	17 (4)		18 (8)	44 (7)	
Chronic diseases, *n (%)*			<0.001			<0.001
None	15 (16)	193 (39)		69 (31)	287 (45)	
1 chronic disease	29 (30)	175 (35)		77 (34)	220 (35)	
2 or more	51 (54)	131 (26)		79 (35)	128 (20)	
Loneliness, *n (%)*			<0.001			<0.001
Rarely or never	60 (59)	389 (78)		113 (50)	444 (70)	
Sometimes or often	41 (41)	109 (11)		112(50)	190 (30)	
Contact with family and friends, *n (%)*			0.08			0.10
Everyday	27 (27)	87 (17)		75 (33)	178 (28)	
Weekly, seldom or not at all	74 (73)	412 (83)		154 (67)	459 (72)	
Contact with acquaintances, *n (%)*			0.07			<0.001
Everyday	9 (9)	19 (4)		23 (10)	18 (3)	
Weekly or monthly	56 (57)	286 (57)		116 (53)	397 (62)	
Seldom or not at all	34 (34)	193(39)		82 (37)	221 (35)	
Alcohol use, *n (%)*			<0.001			<0.001
Weekly	46 (44)	289 (58)		64 (29)	232 (37	
Monthly or less	31 (30)	152 (31)		105 (47)	327 (51)	
Not at all	28 (27)	57 (11)		53 (24)	77 (12)	
Current smokers, *n (%)*	<5 (1)	53 (11)	<0.001	5 (2)	60 (9)	<0.001
Physical activity times per week	2.2	3.1	<0.001	2.1	2.6	<0.001
SF-36: PCS, mean (SD)	40.6 (11.5)	46.4 (9.1)	<0.001	40.2(10.3)	43.7 (10.6)	<0.001
SF-36: MCS, mean (SD)	53.6 (10.5)	55.5 (8.3)	0.06	50.9(10.5)	54.0 (9.9)	<0.001

Data are presented as the mean or *n* (%). SF-36: PCS = physical component summary score. SF-36: MCS = mental component summary score. * Mann-Whitney’s U-test for continuous variables due to non-normality. The chi-squared (χ^2^) test for categorical variables and Fisher’s exact test for dichotomous categorical variables.

**Table 2:** Mean values for the eight SF-36 subscales and physical and mental summary scores among men and women in the two housing groups using the general linear model (GLM).

		Men					Women				
SF-36 Subscales	Model	Senior House		Community-Dwelling			Senior House		Community-Dwelling		
		Mean	S.E.	Mean	S.E.	*p*-Value	Mean	S.E.	Mean	S.E.	*p*-Value
Physical Function	Model 1	67.0	4.5	79.9	1.1	0.001	69.9	2.1	69.9	1.0	0.995
	Model 2	62.6	4.5	80.9	1.0	<0.001	68.6	2.1	70.3	1.0	0.493
General Health	Model 1	62.3	2.4	63.1	0.9	0.772	61.7	1.5	62.5	0.8	0.679
	Model 2	59.6	2.6	63.8	0.9	0.158	61.4	1.6	62.5	0.9	0.599
Bodily pain	Model 1	72.6	3.0	73.6	1.0	0.767	67.2	2.0	63.9	1.1	0.190
	Model 2	68.9	3.2	74.3	1.0	0.134	66.7	2.1	63.8	1.1	0.272
Physical Role	Model 1	70.9	4.9	71.6	1.8	0.897	64.6	3.4	62.7	1.8	0.648
	Model 2	63.7	5.1	73.2	1.8	0.098	62.8	3.5	63.4	1.7	0.888
Mental Health	Model 1	79.6	1.9	82.6	0.7	0.149	76.3	1.4	79.0	0.7	0.087
	Model 2	78.7	2.0	82.9	0.7	0.073	76.7	1.4	78.8	0.7	0.206
Social function	Model 1	82.7	2.6	89.5	0.9	0.019	80.1	1.8	83.9	1.0	0.088
	Model 2	80.3	2.8	90.1	0.9	0.002	80.3	1.9	83.6	1.0	0.153
Vitality	Model 1	69.4	2.3	70.9	0.9	0.559	63.1	1.6	65.2	0.8	0.261
	Model 2	68.1	2.5	71.1	0.9	0.281	63.7	1.6	64.9	0.8	0.536
Emotional Role	Model 1	84.9	3.6	82.9	1.5	0.635	78.6	2.9	74.7	1.6	0.277
	Model 2	83.9	3.7	83.2	1.5	0.879	77.8	3.1	75.1	1.5	0.465
PCS	Model 1	43.4	1.6	45.9	0.5	0.153	43.3	0.9	42.7	0.5	0.582
	Model 2	41.1	1.6	46.4	0.4	0.003	42.7	1.0	42.9	0.5	0.898
MCS	Model 1	55.1	1.2	55.2	0.4	0.916	52.4	0.8	53.6	0.4	0.214
	Model 2	55.2	1.3	55.2	0.4	0.978	52.9	0.8	53.4	0.4	0.560

Model 1 adjusted for age. Model 2 adjusted for age, educational attainment, marital status and economic situation. S.E. = standard error, MCS = mental component summary score, PCS = physical component summary score.

**Table 3:** Senior house residents’ odds for loneliness using binary logistic regression analysis and for social contacts using ordinal logistic regression analysis. Community-dwelling older people as a reference group.

	Model 1			Model 2			Model 3		
Variables	OR	95% CI	*p*-Value	OR	95% CI	*p*-Value	OR	95% CI	*p*-Value
**Male**									
Loneliness	2.32	1.20–4.48	0.012	1.54	0.73–3.27	0.26	1.28	0.59–2.77	0.54
Social contacts:									
Family and friends	1.22	0.63–2.34	0.56	1.08	0.54–2.18	0.83	0.88	0.40–1.94	0.75
Acquaintances	1.39	0.79–2.43	0.25	1.08	0.59–1.97	0.80	1.11	0.57–2.17	0.76
**Female**									
Loneliness	2.01	1.33–3.05	0.001	1.70	1.08–2.67	0.021	1.67	1.06–2.63	0.027
Social contacts:									
Family and friends	1.01	0.61–1.56	0.97	0.85	0.54–1.36	0.51	0.87	0.54–1.39	0.56
Acquaintances	1.14	0.76–1.72	0.57	1.03	0.67–1.59	0.95	1.01	0.65–1.56	0.98

Model 1 adjusted for age, model 2 adjusted for age, educational attainment, marital status and economic situation and model 3 adjusted for age, educational attainment, marital status, economic situation and chronic diseases. OR = odds ratio, CI = confidence interval.

## Data Availability

The datasets generated during and/or analysed during the current study are not publicly available due to sensitive data, but survey data are available from the corresponding author on reasonable request.
